# The effect of different irrigation and disinfection methods on post-operative pain in mandibular molars: a randomised clinical trial

**DOI:** 10.1186/s12903-022-02651-y

**Published:** 2022-12-13

**Authors:** Tuna Kaplan, Sema Sönmez Kaplan, Güzide Pelin Sezgin

**Affiliations:** Department of Endodontics, Faculty of Dentistry, Biruni University, Istanbul, Turkey

**Keywords:** Diode laser, Sonic irrigation activation, Post-operative pain, Symptomatic apical periodontitis

## Abstract

**Background:**

To examine post-operative pain (PP) after conventional irrigation and sonic activation methods, with and without laser disinfection in mandibular molars.

**Methods:**

Eighty patients with symptomatic apical periodontitis were included in this randomized clinical study. There were four study groups. In group 1, conventional irrigation only was applied. In group 2, a sonic irrigation activation system (EDDY (VDW, Munich, Germany)), was applied. In groups 3 and 4, irradiation with a 980-nm diode laser was performed, following irrigation with the conventional method and sonic irrigation activation system, respectively. The patients were instructed to record their PP and analgesic intake using a numerical rating scale 8, 24, 48 h and 7 days post-procedure. A chi-square test, Fisher’s exact chi-square test and Fisher–Freeman–Halton exact test were used to assess qualitative data. Inter-group and intra-group parameters were assessed using the Kruskal–Wallis test and Wilcoxon’s test at a significance level of *p* < 0.05.

**Results:**

There was no statistically significant difference among the groups in terms of age, sex, pre-operative pain, PP and analgesic intake (*p* > 0.05).

**Conclusions:**

The use of sonic irrigation activation system in the final irrigation protocol and irradiation with the 980-nm diode laser did not significantly reduce PP levels and analgesic intake.

**Supplementary Information:**

The online version contains supplementary material available at 10.1186/s12903-022-02651-y.

## Background

Post-operative pain (PP) is a common occurrence after endodontic therapy, with a reported incidence of between 3 and 58% [1]. Several chemical, mechanical and microbial factors associated with root canal treatment are potential sources of PP [1]. Apical periodontitis is a frequent consequence of endodontic infection. According to previous studies, the possibility of healing in the absence of apical periodontitis was 10–15% higher than in the presence of apical periodontitis [2, 3]. The aim of root canal therapy in cases of apical periodontitis is to ensure a microorganism-free environment in the root canal system and periodontium [4]. Two primary factors, the complexity of the root canal system and the feature of the microbial flora, affect the success of root canal therapy [5]. Irrigation plays an important role in disinfecting the root canal and removing bacteria in complex root canal systems, as irrigation solutions can access areas that instruments may be unable to reach during mechanical preparation [6]. Due to the complexity of the root canal system, irrigation solutions alone using conventional needle irrigation may be insufficient [7]. Several irrigation activation systems, such as agitation by gutta-percha cones and brushes, negative pressure devices, laser systems and sonic/ultrasonic devices, have been suggested to increase the disinfection effectiveness in root canal systems [8].

Recently, 980-nm diode laser has been used in endodontic treatment to disinfect root canals [9], with studies reporting that it is a helpful adjunct to conventional irrigation methods due to its disinfection capability [10, 11]. Several studies have reported that laser irradiation may result in decreased PP [12, 13]. EDDY (VDW, Munich, Germany) is a sonic irrigation activation system. It has a flexible 25/04 polyamide tip, powered at a frequency of 6000 Hz by an air scaler. In addition to having the disinfection effectiveness of passive ultrasonic irrigation, the three dimensional motion of the device induces cavitation and acoustic streaming [14]. A previous study noted that the EDDY sonic irrigation activation system decreased PP after root canal treatment in cases of symptomatic irreversible pulpitis [15].

To the best of our knowledge, no studies have investigated the efficiency of the EDDY sonic irrigation activation system in reducing PP severity in teeth with symptomatic apical periodontitis. In addition, there is a knowledge gap in endodontic literature regarding clinical trials on PP in symptomatic non-vital molar teeth. Therefore, the aim of this study was to examine PP severity after using the conventional irrigation method and EDDY, with and without laser disinfection in mandibular molars diagnosed with symptomatic apical periodontitis. The null hypothesis of the present study was that there would be no statistically significant difference in PP levels according to the irrigation and disinfection procedures used.

## Materials and methods

### Sample size

The sample size was calculated based on that obtained in a previous study using G*Power 3.1 (Heinrich Heine University, Dusseldorf, Germany) software [16]. The main study protocol was the same as that in the previous study. The power calculation demonstrated that to achieve an effect size of 0.81 and power of 80% at a p level of 0.05, the minimum sample size in each group was 16 patients. Due to the possibility of dropouts during the treatment or follow-up stages, 20 patients were included in each group, resulting in 80 patients in total in this study.

### Eligibility criteria

The present study was a parallel randomized controlled trial, with an allocation ratio of 1:1. The study complied with the CONSORT guidelines (Additional file 1). In total, 436 patients who visited the Endodontics Department of the Faculty of Dentistry of Biruni University between December 2020 and June 2021 were invited to participate in this study. After obtaining ethical committee approval from the ethics committee of Biruni University (2015-KAEK-43-20-05), 80 patients who fulfilled the inclusion criteria were included in this study. The study was conducted in accordance with the Declaration of Helsinki.

The inclusion criteria were as follows:

1. Healthy adult patients aged between 18 and 65 years
2. Moderate to severe pain (4–10) according to a numerical rating scale
3. Mandibular molar teeth diagnosed with symptomatic apical periodontitis (a painful response during mastication and/or percussion or palpation) and with a negative response to cold test.

The exclusion criteria were:

1. Pregnancy or lactation
2. Taking analgesics or anti-inflammatory drugs 12 h before the treatment
3. Taking antibiotics in the month prior to the treatment
4. Teeth with a PAI score of 3, 4 or 5, calcified root canals, moderate or severe root curvatures in the apical third, greater than grade I mobility and a 4 mm pocket depth, non-restorable damage, occlusal trauma and previous root canal treatment or an open apex
5. Allergic to articaine or non-steroid anti-inflammatory drugs

**Fig. 1:**
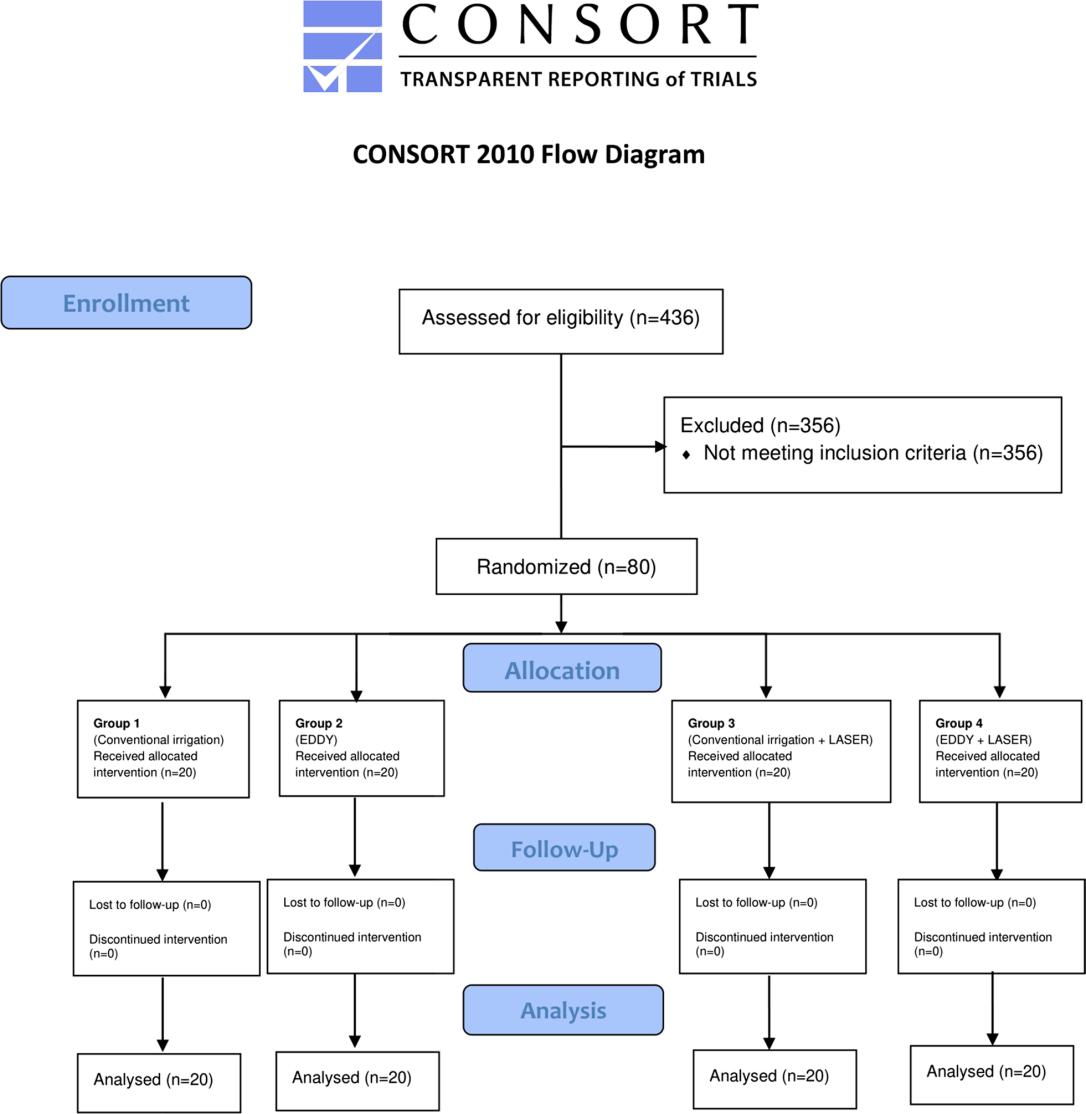
CONSORT 2010 Flow Diagram for randomized clinical trials

All the patients were informed in detail about the benefits and risks of the treatment, and all signed a written consent form. An operator blinded to the study protocol allocated the 80 patients into four groups according to the disinfection and irrigation types (Fig. 1). Randomization was done using computer software (http://www.random.org/). Slips of paper with the group number (1–4) were placed into sealed opaque envelopes to be selected by the patients and opened by the operator before the endodontic treatment. The patients were informed about the research and the devices that would be used, but they were not given information about the group allocation.

### Root canal treatment procedure

All root canal treatment procedures were performed in two visits by a single experienced endodontist. At the first visit, the pre-operative pain scores of each patient were documented. Pain levels were scored from 0 to 10, with 0 denoting no pain and scores of 1–3, 4–6 and 7–10 denoting mild, moderate and severe pain, respectively [17]. Inferior alveolar nerve anaesthesia was performed using articaine 4% solution with epinephrine (Ultracaine D-S forte; Hoechst-Marion Roussel, Frankfurt, Germany) in a concentration of 1:200.000. After rubber-dam isolation was properly executed, endodontic access cavity preparation was done using carbide burs. The working length (WL) was determined using a stainless steel hand K-file size #15 (VDW, Munich, Germany) with an electronic apex locator (ProPex Pixi; Dentsply Sirona, Ballaigues, Switzerland) and then confirmed by an intra-oral periapical radiograph. Root canal preparation was performed using a VDW.ROTATE Ni–Ti file system (VDW, Munich, Germany) with X-Smart Plus Endomotor (Dentsply Maillefer, Ballaigues, Switzerland) at a speed of 350 rpm and 2 N/cm up to 25/0.06 in mesial canals and 35/0.06 in distal canal in all cases. Files were used to reach the WL in all canals by performing with an up and down movement 10 times, for each canal. During root canal preparation, 2 ml of 5.25% sodium hypochlorite (NaOCl) using a 31-gauge double side-port needle (NaviTip; Ultradent, South Jordan, UT) was applied between every two successive files. Final irrigation was then performed in each group.

### Group 1 (conventional irrigation)

After finishing the mechanical instrumentation, each root canal was irrigated with 5 ml of 5.25% NaOCl using a 31-gauge needle positioned 2 mm shorter than the WL. To remove the smear layer, 5 ml of 17% ethylenediaminetetraacetic acid (EDTA) was used in each canal for 1 min, and 5 ml of saline was then administered to neutralize all the residues.

### Group 2 (EDDY)

In group 2, the root canals were irrigated by 2 ml of 5.25% NaOCl agitation for 20 s three times with the EDDY tip positioned 2 mm shorter than the WL. Subsequently, the EDDY tip was activated with short pumping movements, and 2 ml of 17% EDTA was activated for 30 s. The final irrigation followed the same procedures as in group 1 [15].

### Group 3 (conventional irrigation and laser irradiation)

In group 3, final irrigation was performed as in group 1 and followed by laser irradiation. During the laser therapy, both the operator and the patient wore eyewear for protection. Laser irradiation was performed using a 980-nm diode laser (Medency Primo 10 W Diode Laser; Vicenza, Italy), coupled with optical fibre (200 µm). The settings were as follows: average power of 1.2 W with a low frequency of 50 Hz and energy of 12 J (each cycle) in pulsed mode, irradiation for 10 s, followed by a 10 s pause, which constituted one cycle. This cycle was applied four times to each dried root canal. The optical fibre tip was located at the WL. The root canals were then slowly (at a speed of 2 mm/s) irradiated from the apical to the coronal portion using a continuous helicoidal movement, with optical fibre tip contacting the root canal walls in one cycle for each power.

### Group 4 (EDDY and laser irradiation)

In group 4, the same protocol was performed as in group 2 and followed by laser irradiation as in group 3.

All the root canals were dried with paper points, and calcium hydroxide (Ca(OH)_2_) paste (Calsin; Karabağlar, Izmir, Turkey) was applied as intra-canal medicament. A piece of sterile cotton was placed in the pulp chamber, and a temporary restorative material (Cavit-G; 3 M ESPE, St Paul, MN) was used to seal the access cavity. The patients received training on how to complete the numerical rating scale sheets at home (Additional file 2), and they were instructed to record their PP scores on these sheets after 8, 12, 24 and 48 h and after 7 days. The patients were advised that they could take 600 mg of ibuprofen every 8 h for pain alleviation if they suffered from severe pain at any point and that they should record the time interval to medication intake.

At the second visit, the Ca(OH)_2_ was removed from the root canals with final irrigation and using a 25/0.06 file in mesial canals and 35/0.06 in distal canals. The root canals were dried after Ca(OH)_2_ paste removal and obturated using the modified single cone technique. Subsequently, the teeth were restored using a temporary restorative material, and the patients were referred for permanent restorations.

### Statistical analysis

Statistical analysis was performed using IBM SPSS Statistics 22 (SPSS, Chicago, IL). A chi-square test, Fisher’s exact chi-square test and Fisher–Freeman–Halton exact test were used to assess qualitative data. The Kolmogorov–Smirnov test and Shapiro–Wilks test were used to verify the assumption of normality. Inter-group and intra-group parameters were assessed using the Kruskal–Wallis test and Wilcoxon test, respectively. The level of significance was set at *p* < 0.05.

## Results

**Fig. 2:**
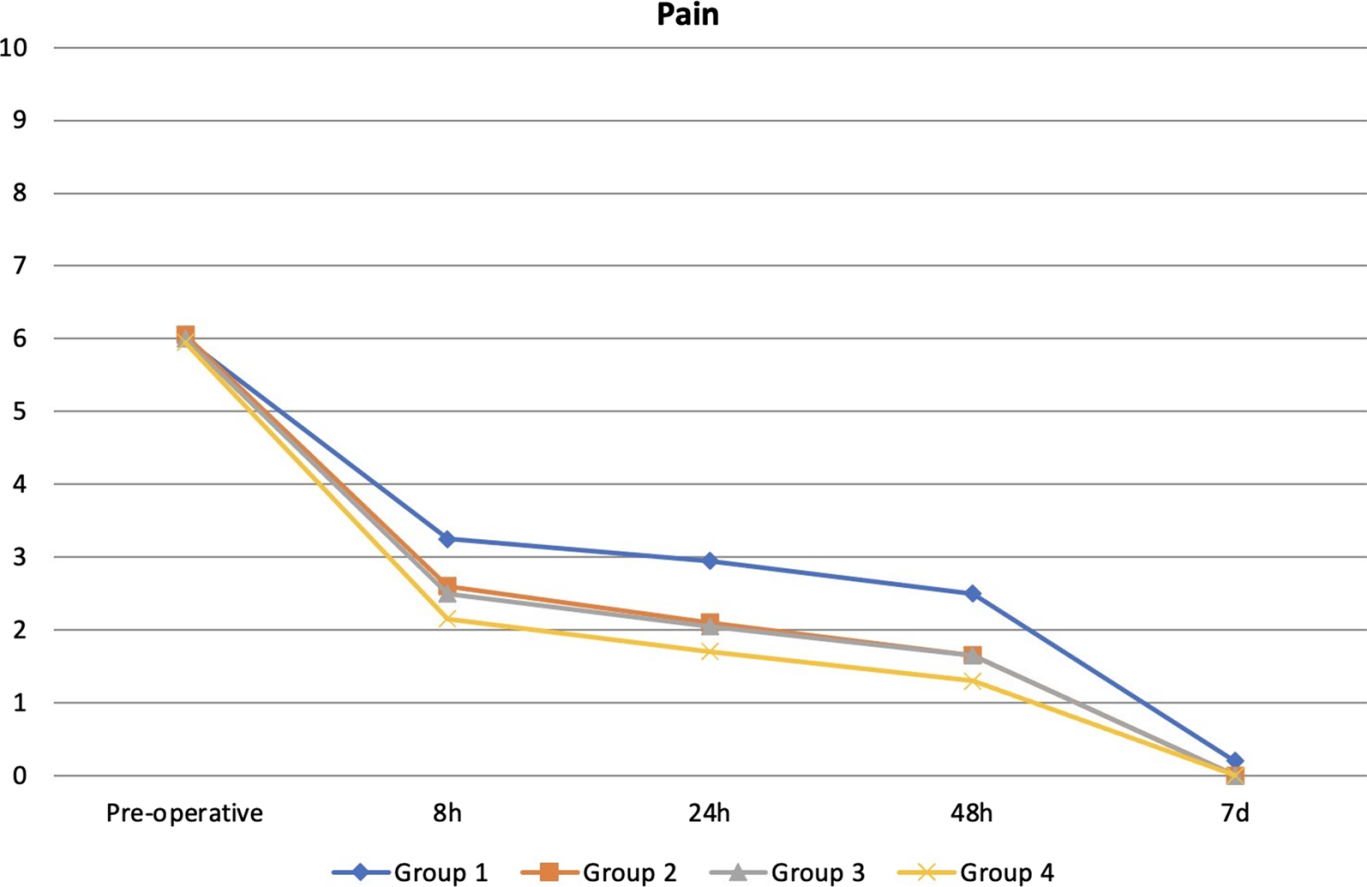
Pain levels in the study groups

Table 1 shows the demographic data for each group. There was no statistically significant difference among the groups in terms of age or sex (*p* > 0.05) (Tables 2 and 3). In terms of pre-operative pain and PP there was no significant difference among groups at the different interval times. (*p* > 0.05) In addition, the decrease was statistically significant between preoperatiove pain and each of the observation periods (*p* < 0.05) (Table 4). There was no significant difference in the PP between males and females as well as among age groups (*p* > 0.05). The decrease in PP levels after 8, 24 and 48 h and after 7 days was statistically significant in all the groups (*p* < 0.05) (Fig. 2). In addition, there was no statistically significant difference in analgesic intake among the groups (*p* > 0.05) (Table 5).

**Table 1:** Demographic data of patients in study groups

	Group 1	Group 2	Group 3	Group 4	*p*
Age Mean±SD (median_)_	35.4 ± 12.89 (35)	32.9 ± 12.96 (32.5)	37.1 ± 13.29 (36.5)	36.3 ± 12.99 (36)	^1^0.663
Sex n (%)					
Male	8(40)	8 (40)	7 (35)	8 (40)	^2^0.984
Female	12 (60)	12 (60)	13 (65)	12 (60)	

^1^Kruskal Wallis Test

^2^Chi-sqare test

**Table 2:** Assessment of pain levels according to age

Pain	18–33	34–49	50–65	^1^*p*
	Mean ± SD (median)	Mean ± SD (median)	Mean ± SD (median)	
Pre-operative	6.14 ± 1.03 (6)	5.87 ± 1.09 (5)	5.92 ± 1.56 (5)	0.340
8 h	2.89 ± 1.91 (3)	2.48 ± 1.98 (3)	2.17 ± 3.01 (0,5)	0.160
24 h	2.51 ± 1.87 (3)	1.94 ± 1.9 (2)	1.92 ± 2.64 (0,5)	0.207
48 h	2.03 ± 1.76 (2)	1.48 ± 1.67 (1)	1.75 ± 2.45 (0,5)	0.342
7 days	0.05 ± 0.33 (0)	0 ± 0 (0)	0.17 ± 0.58 (0)	0.294
Pre-op-8 h ^2^*p*	0.000*	0.000*	0.002*	
Pre-op-24 h ^2^*p*	0.000*	0.000*	0.002*	
Pre-op-48 h ^2^*p*	0.000*	0.000*	0.002*	
Pre-op-7 days ^2^*p*	0.000*	0.000*	0.002*	

^1^Kruskal Wallis test

^2^Wilcoxon sign test

^*^p < 0.05

**Table 3:** Assessment of pain levels according to sex

Pain	Male	Female	^1^*p*
	Mean ± SD (median)	Mean ± SD (median)	
Pre-operative	6.10 ± 1.08 (6)	5.94 ± 1.18 (5)	0.390
8 h	2.77 ± 1.91 (3)	2.53 ± 2.26 (3)	0.469
24 h	2.29 ± 1.87 (2)	2.14 ± 2.10 (2)	0.613
48 h	1.84 ± 1.75 (2)	1.73 ± 1.90 (1)	0.628
7 days	0.06 ± 0.36 (0)	0.04 ± 0.29 (0)	0.742
Pre-op-8 h ^2^*p*	0.000*	0.000*	
Pre-op-24 h ^2^*p*	0.000*	0.000*	
Pre-op-48 h ^2^*p*	0.000*	0.000*	
Pre-op-7 days ^2^*p*	0.000*	0.000*	

^1^Mann Whitney U test

^2^Wilcoxon sign test

^*^p < 0.05

**Table 4:** Pain level distribution in the study groups pre-operative and at 8 h, 24 h, 48 h and 7 d after treatment

Pain	Group 1	Group 2	Group 3	Group 4	^1^*p*
	Mean ± SD (median)	Mean ± SD (median)	Mean ± SD (median)	Mean ± SD (median)	
Pre-operative	6 ± 1.52 (5)	6.05 ± 1.1 (6)	6 ± 0.92 (6)	5.95 ± 1 (5.5)	0.889
8 h	3.25 ± 2,69 (3)	2.6 ± 2.01 (3)	2.5 ± 1.79 (3)	2.15 ± 1.87 (1.5)	0.720
24 h	2.95 ± 2.5 (3)	2.1 ± 1.89 (2)	2.05 ± 1.7 (2)	1.7 ± 1.75 (1)	0.475
48 h	2.5 ± 2.37 (2)	1.65 ± 1.66 (1.5)	1.65 ± 1.53 (1.7)	1.3 ± 1.56 (0.5)	0.409
7 days	0.2 ± 0.62 (0)	0 ± 0 (0)	0 ± 0 (0)	0 ± 0 (0)	0.108
Pre-op-8 h ^2^*p*	0.000*	0.000*	0.000*	0.000*	
Pre-op-24 h ^2^*p*	0.000*	0.000*	0.000*	0.000*	
Pre-op-48 h ^2^*p*	0.000*	0.000*	0.000*	0.000*	
Pre-op-7 days ^2^*p*	0.000*	0.000*	0.000*	0.000*	

^1^Kruskal Wallis test

^2^Wilcoxon sign test

^*^p < 0.05

**Table 5:** Comparison of groups in relation to analgesic use

Analgesic use	Group 1	Group 2	Group 3	Group 4	*p*
	n (%)	n (%)	n (%)	n (%)	
8 h	13 (65)	12 (60)	12 (%60)	9 (45)	0.606
24 h	11 (55)	9 (45)	9 (45)	8 (40)	0.812
48 h	9 (45)	6 (30)	7 (35)	5 (25)	0.581
7 days	0 (0)	0 (0)	0 (0)	0 (0)	–

Chi-square test

## Discussion

An important goal in root canal treatment is to prevent PP. During chemomechanical preparation using conventional irrigation and disinfection methods, debris and the extrusion of irrigation agents from the apical foramen can cause PP. To the best of our knowledge, no study has evaluated PP levels in patients in whom the EDDY irrigation activation system, in combination with disinfection using a 980-nm diode laser, was used. Therefore, the present study aimed to compare the effects of various disinfection and irrigation procedures on PP in mandibular molars diagnosed with symptomatic apical periodontitis.

Patients who had used analgesics for reasons not associated with root canal treatment were not included in the present study to reduce pre-operative factors that could affect the results. In addition, in this study, the population was restricted to patients with no systemic disorders. Patients who might have suffered from diffuse pain were also excluded to eliminate pre-operative factors that could affect the results. Mandibular molars were selected for this study, as the highest prevalence of apical periodontitis has been reported in this tooth type [18, 19]. Root canal treatment was performed in two visits instead of a single visit so that the PP expected as a result of obturation [12] would not effect the study results.

In this study, irrigation was performed using 5.25% NaOCl which showed more efficacy compared to 2.5% [20]. During irradiation with the 980-nm laser, the pulsed mode was used by activating the optical fibre from the apical to coronal slowly with the aim of transferring the beam into the root canal homogeneously. Morsy et al. and Silva Garcez et al. reported that an optical fibre transferred the beam into the root canal homogeneously, providing a more effective photoreaction and higher antibacterial reaction [21, 22].

Many scales have been used to evaluate PP in previous studies [12, 15, 21]. Similar to these studies, we assessed PP levels in this study using a numerical pain rating scale, a segmented numeric version of visual analog scale (VAS), which is easily understood by patients and provides accurate findings [15, 16, 23, 24]. To ensure the reliability of the documented PP levels, prior to the study, the patients were instructed how to use the scale to score their pre-operative pain levels.

The findings obtained from periapical radiographs and electronic apex locators do not always correspond with each other. Over-instrumentation during chemomechanical preparation can cause extrusion of debris to the periapex [25, 26]. Therefore, it is essential to determine the WL accurately to avoid causing PP. In the present study, both periapical radiography and electronic apex locators were used to determine the WL and enhance the reliability and validity of the findings.

In the present study, there was no statistically significant difference among the patient groups in terms of age, sex and pre-operative pain levels. Similarly, Gündoğar et al., who examined PP after different irrigation techniques, detected no statistical significant association among the patient groups in terms of age, sex or pre-operative pain levels [15].

Thus far, no studies have focused on the effects of using both the EDDY (VDW) and a 980-nm diode laser on PP levels. Therefore, the findings of the present study cannot be directly compared to those of other studies. Only one study has examined the effects of the EDDY (VDW) on PP [15]. According to our results, there was no statistically significant difference in PP among the patient groups at any of the documented times. Therefore, the null hypothesis of this study was accepted. In contrast to our findings, Gündoğar et al. reported that PP levels in a patient group in which the EDDY (VDW) was applied were significantly lower in comparison with that in patient groups treated with other irrigation methods in the 24-h post-procedure period [15]. This inconsistency may be caused by the difference between the diagnoses of the patients in the two studies.

In this study, none of the patients experienced severe pain or swelling after endodontic treatment, and PP levels were mostly mild in all the groups. Generally, analgesic intake is related to the PP level. In the present study, the patients were advised that they could take 600 mg of ibuprofen for severe PP because of its dose-dependent activity. There was no significant difference in analgesic medication use among the patient groups in this study. This result is in accordance with that reported in some studies [12, 15] and in contrast to that found in other studies [27, 28]. Genç Şen et al. [27] and Arslan et al. [28] assessed PP in endodontic retreatment cases while Kaplan et al. [12] and Gündoğar et al. [15] did not assess retreatment cases as in the present study. These differences might be the possible explanation of these inconsistencies among the results of the studies.

In contrast to our results, other studies reported significantly lower PP levels in a diode laser group than in what other groups at all post-procedure follow-up times [21, 29, 30]. The fact that cases of chronic periapical lesions and single-rooted teeth were not included in this study, may be a reason for not significantly lower PP levels in laser group compared to other groups unlike other studies. According to Tuner et al., after root canal treatment in chronic cases of apical periodontitis, the situation turns symptomatic when the healing begins as a response of the immune system, thereby leading to an increased risk of PP [31]. However, they reported that patients in whom the root canal treatment involved diode laser disinfection did not experience PP [31]. The difference between our study results and those of the other studies may be explained by our study including only root canal treatment in symptomatic apical periodontitis cases. On the other hand, some studies reported that the risk of PP is associated with the pressure produced in the fluids within the root canal when laser irradiation is performed, the energy used and the position of the optical fibre [32, 33]. Furthermore, PP may be linked to laser activated irrigation extruding more debris from the apical foramen in comparison to conventional irrigation techniques [34]. According to the results of the present study, PP levels dramatically diminished after 48 h in all the groups. A systemic review noted that PP levels after 24 h decreased from 40 to 11% in the 7 days [35].

Although we took steps to ensure standardization in terms of patients and cases in the present study, some factors might have influenced PP levels. As reported previously, anxiety before endodontic treatment and tissue trauma caused by the anaesthesia and rubber-dam procedure may cause PP [36]. Moreover, patients’ perceptions of PP are very subjective and the use of Ca(OH)_2_ intracanal medication can mask the effect of irrigation, agitation and disinfection protocols. To reduce the likehood of the aforementioned factors influencing the results, this study was performed with a relatively large sample size in a real clinical setting.

## Conclusion

At present, PP varies markedly among patients after root canal treatment. Advances in endodontic irrigation-disinfection techniques may be important for easy, effective and minimally invasive root canal treatment to reduce PP. In the present study, the use of sonic irrigation activation system in the final irrigation protocol and irradiation with the 980-nm diode laser did not significantly reduce PP levels and analgesic intake. Therefore, different sonic irrigation activation systems or laser devices should be considered for further clinical studies.

## Supplementary Information

**Additional file 1.** CONSORT 2010 Checklist.

**Additional file 2.** Numerical rating scale.

## Data Availability

The datasets generated during and analyzed during the current study are not publicly available due to the protocol submitted to the Ethics Committee of University of Biruni but are available from the corresponding author on reasonable request.
